# Supplementary material for: Single Nucleotide Polymorphism in the IL17A Gene Is Associated with Interstitial Lung Disease Positive to Anti-Jo1 Antisynthetase Autoantibodies
Source: Life (Basel). 2021 Feb 23;11(2):174. doi: 10.3390/life11020174 (PMC7926454; doi:10.3390/life11020174)
Supplement: Supplementary file 1 [file life-11-00174-s001.pdf]

Single nucleotide polymorphism in the *IL17A* gene is associated with interstitial lung disease positive to anti-Jo1 antisynthetase autoantibodies

Marco Antonio Ponce-Gallegos[1], Montserrat I. González-Pérez[2], Mayra Mejía[2], Karol J. Nava-Quiroz[1], Gloria Pérez-Rubio[1], Ivette Buendía-Roldán[3], Espiridión Ramos-Martínez[4], Jorge Rojas-Serrano[2]*, Ramcés Falfán-Valencia[1]*

Supplementary information

Immunoblot test principle

In the immunoblot, antigens coated on membranes are used as a solid phase to detect specific antibodies in patient samples. The test performance is either manual, semi-, or fully automated. If a sample contains specific antibodies, these bind to the membrane-bound antigens. In the next step, an alkaline phosphatase (AP) labeled antibody (conjugate) is added, which binds to the specific antibodies. The alkaline phosphatase catalyzes a color reaction with the subsequently added nitro blue tetrazolium chloride/5-Bromo-4-Chloro-3-indolyl phosphate (NBT/BCIP). If specific antibodies are present in the patient sample, a dark line appears at the respective antigen position. The intensity of the resulting staining is proportional to the antibody concentration in the sample.

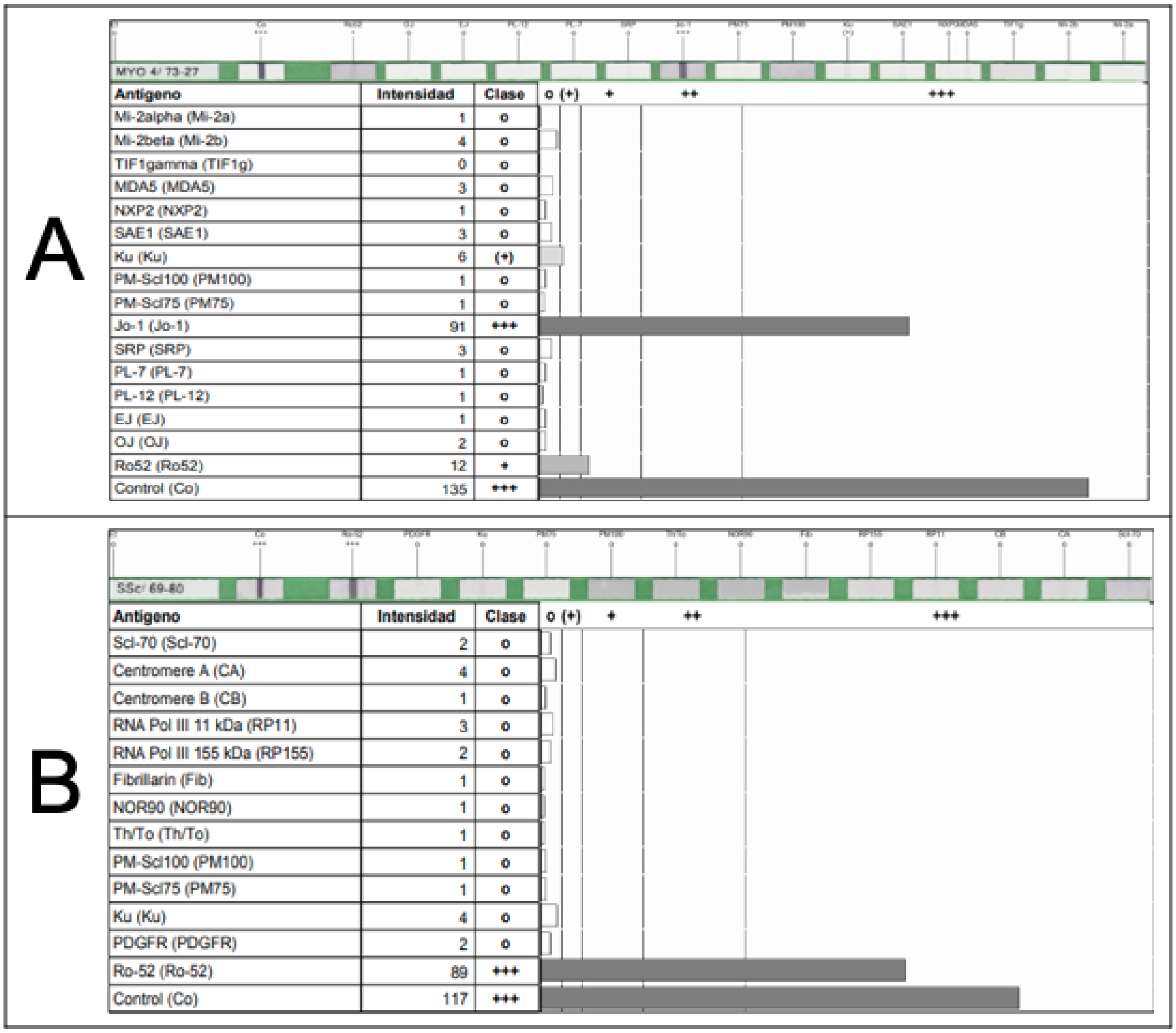

A

MYO 4/ 73-27

Antigeno	Intensidad	Clase
Mi-2alpha (Mi-2a)	1	o
Mi-2beta (Mi-2b)	4	o
TIF1gamma (TIF1g)	0	o
MDA5 (MDA5)	3	o
NXP2 (NXP2)	1	o
SAE1 (SAE1)	3	o
Ku (Ku)	6	(+)
PM-Scl100 (PM100)	1	o
PM-Scl75 (PM75)	1	o
Jo-1 (Jo-1)	91	+++
SRP (SRP)	3	o
PL-7 (PL-7)	1	o
PL-12 (PL-12)	1	o
EJ (EJ)	1	o
OJ (OJ)	2	o
Ro52 (Ro52)	12	+
Control (Co)	135	+++

B

SSc/ 69-80

Antigeno	Intensidad	Clase
Scl-70 (Scl-70)	2	o
Centromere A (CA)	4	o
Centromere B (CB)	1	o
RNA Pol III 11 kDa (RP11)	3	o
RNA Pol III 155 kDa (RP155)	2	o
Fibrillarin (Fib)	1	o
NOR90 (NOR90)	1	o
Th/To (Th/To)	1	o
PM-Scl100 (PM100)	1	o
PM-Scl75 (PM75)	1	o
Ku (Ku)	4	o
PDGFR (PDGFR)	2	o
Ro-52 (Ro-52)	89	+++
Control (Co)	117	+++

Figure S1. Two examples of the immunoblots. (A) Immunoblot showing the Jo1 positive autoantibody. (B) Immunoblot showing the Ro52 positive autoantibody.

Table S1. Allele and genotype frequencies of *IL17A* SNPs evaluated in anti-Ro52+ and anti-Ro52- ASSD patients.

Model	Anti-Ro52+		Anti-Ro52-		***p*-value**
	n= 61	F (%)	n= 59	F (%)	
			**rs2275913**		
**Full-Genotypes**					
GG	43	70.49	38	64.41	0.76
GA	16	26.23	18	30.51	0.60
AA	2	3.28	3	5.08	0.62
**Alleles**					
G	102	83.61	94	79.66	0.43
A	20	16.39	24	20.34	
			**rs8193036**		
**Full-Genotypes**	n= 60	F (%)	n= 55		
TT	32	53.33	34	61.82	0.36
TC	26	43.33	16	29.09	0.11
CC	2	3.33	5	9.09	0.20
**Alleles**					
T	90	75	84	76.36	0.81
C	30	25	26	23.64	

The p-value <0.05 was considered as significative.

Table S2. Allele and genotype frequencies and genetic models of *IL17A* SNPs evaluated in anti-Ro52+ ASSD patients and HS.

Model	Anti-Ro52+		HS		*p*-value	OR	CI 95%
	n= 61	F (%)	n = 340	F (%)			
**rs2275913**							
**Full-Genotypes**							
GG	43	70.49	243	71.47	0.88	0.95	0.52-1.73
GA	16	26.23	92	27.06	0.89	0.96	0.52-1.78
AA	2	3.28	5	1.47	0.32	2.27	0.43-11.98
**Alleles**							
G	102	83.61	578	85.00	0.69	0.90	0.53-1.52
A	20	16.39	102	15.00		1.11	0.66-1.88
**Dominant**							
GG	43	70.49	243	71.47	0.88	0.95	0.52-1.73
GA+AA	18	29.51	97	28.53		1.05	0.58-1.91
**Recessive**							
GG+GA	59	96.72	335	98.53	0.32	0.44	0.08-2.32
AA	2	3.28	5	1.47		2.27	0.43-11.98
**rs8193036**							
**Full-Genotypes**	n= 60	F (%)	n = 343	F (%)			
TT	32	53.33	199	58.02	0.50	0.83	0.48-1.43
TC	26	43.33	125	36.44	0.31	1.33	0.76-2.33
CC	2	3.33	19	5.54	0.48	0.59	0.13-2.59
**Alleles**							
T	90	75	523	76.24	0.77	0.94	0.60-1.47
C	30	25	163	23.76		1.10	0.68-1.68
**Dominant**							
TT	32	53.33	199	58.02	0.50	0.83	0.48-1.43
TC+CC	28	46.67	144	41.98		1.21	0.70-2.10
**Recessive**							
TT+TC	58	96.67	324	94.46	0.48	1.70	0.39-7.50
CC	2	3.33	19	5.54		0.59	0.13-2.59

HS: Healthy subjects; P-value <0.05 was considered as significative.
